# Advances in Imaging and Physiology-Guided Personalized Care in Acute Respiratory Distress Syndrome

**DOI:** 10.3390/medicina62020420

**Published:** 2026-02-23

**Authors:** Lucas Rodrigues Moraes, Pedro Leme Silva, Denise Battaglini, Patricia Rieken Macedo Rocco

**Affiliations:** 1 Laboratory of Pulmonary Investigation, Carlos Chagas Filho Institute of Biophysics, Federal University of Rio de Janeiro, Rio de Janeiro 21941-902, RJ, Brazil; lucasmoraes.ft@gmail.com (L.R.M.); pedroleme@biof.ufrj.br (P.L.S.); 2 Department of Surgical Sciences and Integrated Diagnostics (DISC), University of Genoa, 16126 Genoa, Italy; battaglini.denise@gmail.com; 3 Anesthesia and Intensive Care, IRCCS Azienda Ospedaliera Metropolitana, 16132 Genoa, Italy

**Keywords:** acute respiratory distress syndrome, mechanical ventilation, oxygenation, driving pressure

## Abstract

Acute respiratory distress syndrome (ARDS) is a heterogeneous inflammatory lung injury marked by increased alveolar–capillary permeability, reduced respiratory system compliance, and impaired gas exchange. Despite advances in supportive care, ARDS remains associated with high mortality. Lung-protective ventilation with low tidal volumes and prone positioning is the cornerstone of treatment. However, these strategies do not fully account for patient-specific physiological variability. Recent guidelines emphasize a more individualized approach to respiratory support. Key elements include limitation of driving pressure, optimized use of high-flow nasal oxygen, and application of bedside tools such as the SpO_2_/FiO_2_ ratio and lung ultrasound. These measures improve diagnosis, monitoring, and physiological assessment at the bedside. This narrative review summarizes current evidence supporting contemporary ventilatory and non-invasive strategies in ARDS. It also examines emerging diagnostic and therapeutic approaches that integrate respiratory physiology into clinical decision-making. Finally, we discuss future directions focused on personalized, physiology-guided management to improve outcomes in patients with ARDS.

## 1. Introduction

Acute Respiratory Distress Syndrome (ARDS) is a severe form of acute non-cardiogenic respiratory failure and remains a major challenge in contemporary intensive care [1,2,3,4,5,6]. It results from diffuse injury to the alveolar-capillary barrier, leading to loss of aerated lung tissue, reduced respiratory system compliance, increased venous mixing and dead space, and severe impairment of gas exchange [2,4,5,7]. Patients who meet diagnostic criteria may differ substantially in lung recruitment, respiratory mechanics, inflammatory burden, and response to ventilatory and adjuvant therapies. Lung-protective ventilation and early prone positioning remain the cornerstone of ARDS management and are supported by robust clinical evidence [1,3,8,9]. However, these approaches alone do not fully explain interindividual differences in respiratory mechanics or disease trajectory. As a result, attention has increasingly turned to physiologically guided and biologically informed strategies, including refined ventilatory targets, advanced bedside assessment tools, and emerging therapies aimed at modulating lung injury and repair [2]. Despite advances in supportive care, ARDS remains associated with substantial and variable mortality, reflecting the heterogeneity in disease severity, underlying etiologies, and physiological presentation [10]. In this context, this narrative review synthesizes current evidence on the diagnosis and treatment of ARDS and examines emerging therapeutic concepts, with particular emphasis on innovations that support individualized and physiology-driven care. 

## 2. Updated Diagnostic Criteria for ARDS

Historically, ARDS has been defined primarily by radiographic abnormalities and the severity of hypoxemia, most assessed using the PaO_2_/FiO_2_ ratio [11,12,13]. Berlin definition formalized this approach and required the presence of positive end-expiratory pressure (PEEP) of at least 5 cmH_2_O at diagnosis [4]. Consequently, patients not receiving positive pressure ventilation were excluded, limiting applicability in early disease stages and in settings with limited access to invasive respiratory support. Recent revisions have sought to address these limitations. Prompted by lessons from the COVID-19 pandemic and by marked global disparities in access to invasive ventilation, arterial blood gas analysis, and advanced imaging, updated definitions broaden ARDS diagnosis to include patients managed with conventional oxygen therapy, high-flow nasal oxygen (HFNO), or non-invasive ventilation (NIV), using validated non-invasive indices. This framework retains the fundamental pathophysiological concepts underpinning the Berlin definition while enhancing diagnostic feasibility and relevance across heterogeneous healthcare settings [3,8]. Although the new classifications increase diagnostic sensitivity and facilitate early identification, specificity may be reduced, increasing the risk of misclassification of other causes of hypoxemic respiratory failure. Furthermore, broader diagnostic definitions may also influence the epidemiology of ARDS, excessively increasing the number of cases and reducing phenotypic homogeneity in interventional clinical trials [5,14]. Therefore, it is possible to conclude that many treatments for ARDS fail due to their heterogeneity [15]. In several previous cohort studies, hypo-inflammatory and hyper-inflammatory subphenotypes were observed, with inverse relationships between mortality and days free from mechanical ventilation [16,17].

Recently, a new classification of ARDS heterogeneity based on oxygenation was presented. These subgroups were named “persistently low,” “gradually increasing”, and “rapidly improving”. These dynamic subgroups are more strongly associated with patient outcomes and allow for clearer differences in treatment regarding PEEP [18]. Furthermore, the Delphi study proposed that the diagnosis of ARDS should consider the following criteria: time of onset, risk factors, PaO_2_/FiO_2_ ratio, infiltrates on chest imaging, and exclusion of alternative diagnoses [19]. Therefore, recognizing ARDS subphenotypes may allow for more appropriate treatment in selected patients.

## 3. High-Flow Nasal Oxygen

Updated definitions seek to extend diagnostic criteria to non-intubated patients. The ESICM 2023 guidelines recognize that patients receiving HFNO may meet diagnostic criteria for ARDS [3]. This concept is further clarified in the 2024 Global Definition, which explicitly includes patients treated with HFNO at flows ≥30 L/min in the presence of significant hypoxemia [8]. In resource-limited settings, the SpO_2_/FiO_2_ ratio ≤315, with SpO_2_ ≤ 97%, is proposed as a practical alternative to arterial blood gas analysis and it is validated surrogate for PaO_2_/FiO_2_, with similar discriminatory capacity [1,8]

Despite these advances, important uncertainties remain. Thresholds for oxygen flow, delivered FiO_2_, duration of HFNO therapy, and criteria for treatment escalation are not clearly defined [3,8]. During the COVID-19 pandemic, a ROX index [the ratio of (SpO_2_/FiO_2_) to respiratory rate] below 4.88 was associated with HFNO failure, but this cutoff was derived from a specific clinical context and has not been validated in broader ARDS populations [20]. At present, HFNO should be viewed primarily as a diagnostic and supportive modality, requiring close monitoring and timely escalation when clinical deterioration occurs.

## 4. Non-Invasive Ventilation

Non-invasive ventilation is well established for acute hypercapnic respiratory failure, particularly in exacerbations of chronic obstructive pulmonary disease, and for cardiogenic pulmonary edema [21,22]. Its role in hypoxemic respiratory failure and ARDS, however, remains uncertain. During the COVID-19 pandemic, the Surviving Sepsis Campaign issued a weak recommendation for NIV use when HFNO failed, reflecting limited evidence and ongoing concerns regarding safety and efficacy [23].

The ESICM 2023 guidelines do not recommend routine use of NIV or CPAP in ARDS but acknowledge that these modalities may be considered under strict clinical supervision. Continuous evaluation of respiratory effort, ventilatory drive, risk of patient self-inflicted lung injury (P-SILI), and hemodynamic status is essential [3]. A recent randomized trial comparing NIV and HFNO showed no reduction in intubation or mortality at seven days in most ARDS patients; only immunocompromised patients with acute hypoxemic respiratory failure failed to benefit from HFNO alone [24].

Recent definitions states that patients undergoing CPAP or NIV with a PEEP of at least 5 cmH_2_O and an SpO_2_/FiO_2_ ratio ≤315, when SpO_2_ is ≤97%, fulfill diagnostic criteria. However, uncertainties persist regarding optimal interfaces, patient selection, and reliable predictors of success. Consistent with prior recommendations, both guidelines [3,8] emphasize that NIV should not delay intubation in the setting of clinical deterioration.

## 5. Advances in Imaging Techniques for ARDS

### 5.1. Chest Computed Tomography

Chest computed tomography (CT) provides a high-resolution structural assessment of the lungs and remains the gold standard imaging modality for characterizing ARDS morphology [1,25,26,27,28]. CT provides a detailed assessment of regional lung aeration, lung weight, and disease distribution, characteristically demonstrating a dorsoventral gradient of opacities driven by gravitational edema and alveolar collapse. Quantitative analysis allows classification of lung compartments by aeration status, distinguishing non-aerated, poorly aerated, normally aerated, and hyperinflated regions, thus delineating the pronounced spatial heterogeneity of ARDS [25,26,27,28,29].

In addition to diagnosis, CT has contributed substantially to the understanding of ARDS subphenotypes, particularly the distinction between focal and non-focal patterns, which has important implications for recruitment capacity, response to PEEP, assessment of mechanical power, and prone positioning [25]. Current definitions continue to recognize bilateral opacities on CT as supportive diagnostic criteria [3,8]. However, the routine use of CT is limited by the need for patient transport, radiation exposure, and restricted availability, especially in resource-limited settings where chest radiography remains the primary imaging tool. Furthermore, CT findings are not specific to ARDS and may overlap with other causes of lung injury, such as pneumonia [26,27]. However, in centers with high resource availability, CT can be used as a tool to guide initial therapy or its escalation [25]. Another limiting factor is that most CT-based evidence comes from studies in invasively ventilated patients. Although recent definitions include non-intubated patients [3,8], the data supporting the use of CT in this population remain largely observational.

### 5.2. Lung Ultrasound

Lung ultrasound (LUS) has emerged as a widely accessible bedside imaging modality that provides real-time information on lung aeration [30,31]. Characteristic findings in ARDS include bilateral B-lines, subpleural consolidations, and pleural line irregularities, reflecting interstitial edema, alveolar flooding, and loss of aeration. These features align with radiographic criteria but do not directly correspond to the concept of “opacities” used in chest radiography or CT [26,31,32,33,34].

LUS is particularly valuable for monitoring dynamic changes in lung aeration. Semi-quantitative approaches, such as the LUS Score [26,31,33,34,35], allow assessment of regional and global aeration and are useful for tracking disease evolution and response to interventions. Recent validation of the LUS-ARDS Score represents an important step toward standardization, demonstrating promising diagnostic performance [34,35]. However, LUS findings are not specific to ARDS and frequently overlap with other conditions, notably pneumonia. Interpretation is operator dependent, and ultrasound cannot detect pulmonary overdistension.

Recent advances in lung ultrasound have enhanced its role in the diagnosis and monitoring in ARDS. A prospectively derived and externally validated lung ultrasound score (the LUS-ARDS score) has shown promising diagnostic accuracy comparable to expert chest radiograph interpretation and may improve objectivity in identifying ARDS at the bedside, with areas under the receiver operating characteristic curve of ~0.80–0.90 across validation cohorts [36]. These findings support an expanding role for lung ultrasound not only in the serial assessment of regional aeration, but also within composite diagnostic strategies to identify bilateral lung involvement and facilitate early ARDS recognition, particularly when conventional imaging such as CT is unavailable or impractical [37].

Consequently, LUS should be regarded primarily as a monitoring and decision-support tool rather than a standalone diagnostic test. Its strength lies in bedside assessment of aeration trends and differentiation between cardiogenic and non-cardiogenic pulmonary edema, while definitive diagnosis requires integration with clinical context and complementary imaging.

### 5.3. Electric Impedance Tomography

Electrical impedance tomography (EIT) is a non-invasive, radiation-free bedside technique that enables continuous monitoring of regional ventilation distribution [25,38,39]. By detecting impedance changes related to variations in lung air content, EIT provides functional images that reflect tidal ventilation and end-expiratory lung volume [38,39,40].

In ARDS, EIT consistently demonstrates reduced ventilation in dependent regions with preferential redistribution toward non-dependent lung zones, a pattern driven by increased lung weight and pleural pressure gradients [38,39]. These features can be dynamically assessed during changes in ventilatory settings, making EIT particularly useful for evaluating recruitment, overdistension, and regional compliance. Importantly, EIT can detect *pendelluft* and other phenomena associated with ventilator-induced lung injury (VILI), supporting individualized ventilatory strategies [40,41,42].

Despite these strengths, EIT does not provide anatomical information, cannot distinguish etiologies of pulmonary edema, and lacks validated diagnostic thresholds for ARDS. Its clinical role is therefore confined to monitoring and optimization of mechanical ventilation rather than diagnosis. Widespread adoption remains limited by cost, availability, and the need for specialized expertise, and current guidelines do not endorse EIT as a diagnostic modality.

Recent evidence suggests that individualized PEEP titration guided by EIT may be particularly advantageous in complicated physiological contexts such as concurrent intra-abdominal hypertension (IAH). A recent clinical study demonstrated that EIT-based PEEP adjustment improved arterial oxygenation in ARDS patients with IAH without producing significant adverse hemodynamic impairment, although the presence of elevated intra-abdominal pressure may moderate the magnitude of the oxygenation response. Such individualized PEEP strategies highlight the capacity of EIT not only to optimize regional ventilation distribution but also to tailor ventilatory settings to specific extracorporeal pressures that influence chest wall mechanics and lung recruitment, further extending the potential role of EIT in physiological PEEP optimization beyond traditional recruitment and overdistension balancing [43].

### 5.4. PET-CT and SPECT

Positron emission tomography–computed tomography (PET-CT) and single-photon emission computed tomography (SPECT) have been used predominantly in research settings to explore ARDS pathophysiology. These techniques provide unique insights into regional inflammation, perfusion heterogeneity, and metabolic activity, revealing that inflammatory processes may extend beyond radiographically consolidated lung regions and contribute to VILI [27,29].

Although these modalities have substantially advanced the conceptual understanding of ARDS, their clinical application is limited by high cost, radiation exposure, limited availability, and lack of bedside feasibility. As a result, PET-CT and SPECT remain investigative tools that inform translational research rather than routine clinical management.

## 6. Therapeutic Strategies in ARDS: From Non-Invasive to Invasive Support

The management of ARDS has shifted from a predominantly invasive, ventilation-centered paradigm to a staged and physiology-driven approach. Contemporary strategies integrate early non-invasive support, individualized mechanical ventilation, and selective use of adjunctive and rescue therapies, with the goal of improving oxygenation while minimizing VILI.

### 6.1. Active Prone Positioning in Non-Invasively Ventilated Patients

Active prone positioning (APP) induces favorable changes in lung aeration, ventilation, and perfusion in spontaneously breathing patients [44,45,46,47,48,49,50,51]. By redistributing lung inflation from dorsal to ventral regions and reducing gravitational gradients in perfusion, APP improves ventilation–perfusion matching and arterial oxygenation [51]. These effects are accompanied by enhanced lung recruitment, attenuation of hypoxic pulmonary vasoconstriction, and beneficial hemodynamic changes, including increased venous return and reduced right ventricular afterload [51] (Supplementary Table S1).

APP promotes caudal diaphragmatic displacement, reducing compression of dependent lung regions, optimizing diaphragmatic geometry, and improving neuromechanical efficiency. It also increases lung compliance by optimizing the distribution of lung stress and strain and promoting a more homogeneous pattern of regional ventilation [52].

Although APP was originally established as a lung-protective strategy in invasively ventilated patients, its use expanded during the COVID-19 pandemic to non-intubated individuals receiving high-flow nasal oxygen or non-invasive ventilation. Available evidence consistently demonstrates improvements in oxygenation and suggests potential reductions in ICU length of stay and intubation rates in selected patients, particularly when APP is applied early and maintained for prolonged daily periods [44,49,50,53].

However, APP is not without risk. Inadequate patient selection, insufficient monitoring, or delayed recognition of clinical deterioration may postpone intubation and increase the risk of patient self-inflicted lung injury. Moreover, although EIT has been explored in patients receiving non-invasive support, technical and interpretative limitations persist [52]. Therefore, APP should be regarded as an adjunctive intervention within a structured and closely monitored respiratory support strategy, rather than a definitive therapy.

### 6.2. Prone Position in Invasively Ventilated Patients

Prone positioning is a cornerstone therapy in patients with moderate to severe ARDS [54,55,56,57,58,59,60,61,62,63,64]. Beyond improving oxygenation, prone positioning redistributes transpulmonary pressures and tidal ventilation, resulting in a more homogeneous distribution of mechanical forces and a reduction in regional overdistension and cyclic collapse. These effects directly mitigate the risk of VILI [63] and reduces mortality [58,59,62].

The survival benefit of early prone positioning was clearly demonstrated in the PROSEVA trial [64], establishing prone positioning as a core component of lung-protective ventilation rather than a rescue intervention. Improvements in oxygenation primarily reflect enhanced ventilation–perfusion matching rather than increased lung recruitment alone.

Studies have examined the optimal duration of prone positioning sessions [59,60]. While prolonged sessions of 24–48 h have been associated with reduced mortality in selected cohorts, conventional 16-h protocols have been linked to more ventilator-free days, less exposure to neuromuscular blockade, and earlier extubation. These findings underscore the importance of balancing physiological benefits with sedation burden and the risk of ICU-acquired weakness.

Physiological monitoring with EIT has provided mechanistic insight into the effects of prone positioning, demonstrating increased dorsal ventilation and perfusion, as well as reductions in driving pressure and mechanical power [54]. However, clinical responses remain heterogeneous. Not all studies have shown consistent benefits in oxygenation or clinical outcomes, reflecting variability in lung morphology, recruitability, and disease severity. Prolonged prone positioning may also reduce staff workload in resource-limited settings, but this pragmatic consideration should not override individualized physiological assessment [59,60] (Supplementary Table S2).

### 6.3. Ventilatory Strategies: Low and Ultra-Low Tidal Volumes

Lung-protective ventilation remains the foundation of ventilatory management in ARDS and is universally recommended by recent international guidelines. Evidence shows that reductions in driving pressure, rather than low tidal volume per se, are independently and better associated with improved survival in ARDS. Tidal volume, PEEP, and respiratory system compliance interact dynamically to determine driving pressure, which more directly reflects the cyclic lung stress experienced by the aerated lung. Accordingly, tidal volume should be viewed as an adjustable ventilatory variable, titrated to reduce driving pressure, rather than as an isolated therapeutic target [1,3,8]. Conventional targets of 4–8 mL/kg predicted body weight (PBW) aim to limit alveolar overdistension and reduce VILI [65,66]. In selected patients, ultra-protective strategies using tidal volumes of 3–4 mL/kg PBW [67,68,69,70,71] and, in extreme cases, 1–2 mL/kg PBW [72] supported or not by extracorporeal carbon dioxide removal (ECCO_2_R) or ECMO have been proposed to further reduce lung stress and strain [67,68,71,72,73].

The rationale for ultra–low tidal volume ventilation derives from the concept of the “baby lung” [7], which emphasizes that functional lung size, rather than body weight, should guide ventilatory settings. Although ultra-protective strategies may reduce driving pressure, consistent reductions in mortality or length of stay have not been demonstrated, and severe hypercapnia with respiratory acidosis remains a major limitation [67]. Randomized trials comparing driving pressure–targeted strategies with conventional lung-protective ventilation have failed to show clear superiority, highlighting the difficulty of translating physiological optimization into clinical benefit [65,69].

Increasing evidence also indicates that PBW is an imperfect surrogate for functional lung size. Observational studies suggest that predicted forced vital capacity and indices of ventilatory efficiency may better capture interindividual variability, particularly in shorter patients, who appear more susceptible to hypoventilation and excessive strain when PBW-based tidal volumes are applied [74]. Approaches that integrate physiological variables such as driving pressure, dead space, or end-expiratory lung volume are promising but remain insufficiently validated for routine clinical use [75,76,77] (Supplementary Table S3).

### 6.4. PEEP Optimization and Alveolar Recruitment

PEEP is essential for preventing alveolar collapse, improving oxygenation, and reducing atelectrauma [78,79]. However, excessive PEEP may cause overdistension, barotrauma, hemodynamic compromise, and worsening VILI [80]. Current guidelines therefore discourage the routine use of aggressive recruitment maneuvers [3,8] and recent data emphasize individualized PEEP selection [80,81,82,83,84,85,86,87].

Chest CT remains the reference standard for assessing lung recruitability [88,89], but its routine use is impractical. Traditional PEEP/FiO_2_ tables have not demonstrated mortality benefit [90], highlighting the limitations of uniform approaches in a highly heterogeneous syndrome. Compliance-based PEEP titration is feasible at the bedside but may expose unselected patients to lung injury [91]. The recruitment-to-inflation (R/I) ratio has been proposed as a tool to estimate the balance between alveolar recruitment and overdistension [85]. Higher R/I values are associated with improved compliance and reduced dynamic strain, suggesting potential utility in identifying patients likely to benefit from higher PEEP [83,87].

EIT has emerged as a valuable bedside tool for guiding PEEP by simultaneously quantifying regional collapse and overdistension [38,40,84,92]. EIT-guided strategies have been associated with reductions in lung collapse, improved mechanical power, and more favorable regional ventilation patterns [80,84,86,92,93], including during prone positioning and ECMO support [94]. Nonetheless, limited availability, cost, and the need for specialized expertise restrict widespread implementation.

In patients with preserved spontaneous breathing, optimizing PEEP may reduce injurious respiratory effort and *pendelluft*. Physiological studies suggest that moderate-to-high PEEP levels may stabilize ventilation distribution, although clinical benefits remain uncertain [92].

Lung ultrasound represents a more accessible alternative. Ultrasound-guided PEEP compared with conventional strategies has reported lower PEEP requirements, improved compliance, and better gas exchange [95]. Given its growing role in ARDS diagnosis, ultrasound-guided PEEP titration warrants further investigation.

Finally, heart–lung interactions during recruitment are increasingly recognized [82,96]. While PEEP may improve left ventricular performance, effects on right ventricular function are variable and depend on recruitability and baseline pulmonary vascular load. These interactions reinforce the need for integrated respiratory and hemodynamic assessment (Supplementary Table S4).

### 6.5. Neuromuscular Blockade as Adjunctive Therapy

Neuromuscular blocking agents (NMBAs) reduce respiratory effort, patient-ventilator asynchrony, and oxygen consumption, thus facilitating lung-protective ventilation and prone positioning in selected patients with severe ARDS [97,98,99,100,101,102,103,104]. Recent findings show that expiratory muscle activity in ARDS can reduce lung volume at the end of expiration, counteracting the effects of applied PEEP [105]. This so-called anti-PEEP effect leads to alveolar collapse and severe lung injury. The use of muscle relaxants can attenuate this mechanism, abolishing the recruitment of expiratory muscles, increasing lung volume, and improving homogeneity in selected patients.

Current guidelines diverge, with some conditional recommendations supporting the short-term use of NMBAs in severe ARDS and others advising against routine application [3,8]. Evidence from randomized trials is conflicting. While the ACURASYS study [98] demonstrated a survival benefit with early and short-term use of NMBAs under deep sedation, the ROSE study [99] found no mortality advantage and reported higher rates of cardiovascular complications and weakness. Observational studies suggest that NMBAs may improve oxygenation and reduce the duration or number of prone positioning sessions, particularly when driving pressures are controlled [106]. However, the increased need for vasopressors and the risk of neuromuscular weakness remain important concerns.

Recently, a reanalysis of the ACURASYS trial [103,104] explored the heterogeneity of the effect of NMBAs. In patients under 60 years, cisatracurium improved survival and reduced the duration of mechanical ventilation. In contrast, patients over 60 showed no clear benefit and were more susceptible to the effects of prolonged paralysis, such as muscle weakness. Furthermore, and contrary to current guidelines, the use of NMBAs resulted in positive outcomes in moderate ARDS.

However, complete neuromuscular blockade is associated with important drawbacks, including diaphragmatic and peripheral skeletal muscle atrophy due to immobility, as well as the need for deep sedation. An alternative strategy is to titrate diaphragmatic activity using partial neuromuscular blockade. In a recent study, rocuronium was adjusted to permit low, but not abolish, respiratory effort, resulting in lower transdiaphragmatic pressure swings [107].

These findings suggest that NMBAs should be reserved for individualized use in ARDS, avoiding their indiscriminate use. There are still gaps to be filled, such as the depth and duration of the block, indicating the need for future randomized clinical trials (Supplementary Table S5).

## 7. The Future of ARDS During Machine Learning Era

Despite advances in protective ventilation and supportive care, ARDS mortality remains high. One major limitation of past clinical trials is the marked biological and physiological heterogeneity of the syndrome, which complicates patient selection and therapeutic targeting [1,3,8,10]. Recent refinements in ARDS definitions have expanded diagnostic tools but may also increase subphenotypic variability.

Machine learning (ML) approaches have emerged as tools to address these challenges by integrating complex clinical, physiological, and imaging datasets [108]. ML algorithms have shown promise in improving diagnostic accuracy through automated analysis of chest radiographs and CT scans, including identification of ARDS subphenotypes [109,110]. Prognostic models incorporating ML have demonstrated improved mortality prediction compared with traditional scoring systems, such as SOFA, in selected cohorts [111,112,113].

Beyond diagnosis and prognosis, ML applications extend to ventilatory management. Algorithms have been developed to assist in PEEP selection and recruitment maneuvers, detect patient–ventilator asynchrony, evaluate responses to prone positioning, estimate mechanical power, and predict ECMO outcomes [108,114,115,116,117]. ML-based tools have also shown utility in predicting successful ventilator weaning and extubation [118].

Nevertheless, most ML models remain retrospective, single-center, and insufficiently validated for routine clinical use. Challenges related to interpretability, generalizability, and integration into bedside workflows must be addressed. ML should be viewed as a decision-support tool that complements, rather than replaces, physiological reasoning and clinical judgment.

## 8. Conclusions

ARDS remains a complex and heterogeneous syndrome that demands individualized, physiology-based management. Established strategies, including lung-protective ventilation and prone positioning, continue to form the foundation of care. Emerging approaches, such as ultraprotective ventilation and advanced imaging, reflect a growing emphasis on personalization rather than uniform treatment algorithms. Machine learning has the potential to refine diagnosis, prognostication, and ventilatory management, but its clinical impact will depend on careful validation and thoughtful integration. Future progress in ARDS will require aligning mechanistic understanding with patient-centered trial design to translate innovation into meaningful clinical benefits.

## Data Availability

No new data were created or analyzed in this study. Data sharing is not applicable to this article.

## Supplementary Materials

The following supporting information can be downloaded at: https://www.mdpi.com/article/10.3390/medicina62020420/s1, Table S1: Randomized and physiological studies evaluating awake prone positioning in non-intubated patients with COVID-19–related acute hypoxemic respiratory failure; Table S2: Prone positioning in patients with ARDS, including those supported with ECMO: clinical studies evaluating duration, timing, physiological effects, and outcomes; Table S3: Summary of randomized and observational studies evaluating driving pressure–guided and ultra-protective ventilation strategies, and extracorporeal support in patients with ARDS; Table S4: Physiological and clinical studies evaluating PEEP titration strategies and advanced monitoring in patients with ARDS; Table S5: Neuromuscular blockade in ARDS: physiological and clinical studies.
