# Supplementary material for: Advances in Imaging and Physiology-Guided Personalized Care in Acute Respiratory Distress Syndrome
Source: Medicina (Kaunas). 2026 Feb 23;62(2):420. doi: 10.3390/medicina62020420 (PMC12942013; doi:10.3390/medicina62020420)
Supplement: Supplementary file 1 [file medicina-62-00420-s001.zip › medicina-4142066-supplementary.pdf]

Supplementary Table S1. Randomized and physiological studies evaluating awake prone positioning in non-intubated patients with COVID-19–related acute hypoxemic respiratory failure.

Author /Year	Study Design	Population	Age (Years)	Ventilatory Strategy	Prone Positioning Exposure	Primary Outcome	Secondary outcome
Rosén et al. 2021 (41)	RCT	75 patients with moderate-to-severe COVID-19 ARDS (36 PP, 39 standard care)	PP: 66 [53–74]; SC: 65 [55–70]	HFNO (PP: 31; SC: 29); NIV (PP: 5; SC: 10). Flow 50 [40–50] L/min. PEEP (NIV): 7 [6–10] vs 8 [6–8] cmH_2O. FiO_2: 0.60 [0.55–0.70] in both groups	PP: 8.5 [5.2–12.2] h/day; SC: 2.6 [0.3–8.1] h/day	No difference in intubation rate	Fewer pressure sores in PP group (6% vs 23%, p=0.032). No differences in NIV use, vasopressors, RRT, ECMO, ventilator-free days, ICU/hospital LOS, or 30-day mortality
Erhmann et al. 2021 (44)	RCT (meta-trial)	1126 suspected or confirmed COVID-19 patients on HFNO; APP (n=567) vs standard care (n=559); P/F <300 or S/F <315	APP: 62 (± 13); SC: 61 (± 14)	HFNO; flow at maximum tolerated; FiO_2 titrated to SpO_2 90–95%	Median 5 h/day	Treatment failure (intubation or death): 40% vs 46%; RR 0.86 [0.75–0.98]	Lower intubation on day 28 (33% vs 40%). No difference in ICU/hospital LOS
Kaur et al. 2021 (43)	Post hoc analysis of RCT	125 COVID-19 ARDS patients on HFNO (<24 h); Early APP (n=92) vs Late APP (n=33)	Overall: 62 (± 12); Early: 61 (± 12); Late: 66 (± 10)	HFNO 40–50 L/min; FiO_2 titrated to SpO_2 90–95%	Early: 5.1 [2.0–9.1] h/day; Late: 3.0 [1.1–5.6] h/day	28-day mortality: 26% vs 45%; p=0.039	-

Alhazzani et al. 2022 (50)	RCT	400 patients with suspected/confirmed COVID-19 requiring ≥40% O_2 (COT, HFNO or NIV); APP (n=205) vs SC (n=195)	APP: 57 (± 12); SC: 58 (± 13)	32 NIV; 88 COT; 280 HFNO. Median FiO_2: 70% [55–90] vs 70% [52–90]	Median 8–10 h/day	Intubation by day 30: 34.1% vs 40.5%; p=0.20	No differences in 60-day mortality, ventilator-free days, ICU- or hospital-free days
Fralick et al. 2022 (COVID-PRONE) (45)	RCT	248 patients with confirmed COVID-19, FiO_2 >50%; PP vs SC	PP: 60 [45–68]; SC: 54 [44–62]	COT or HFNO (flow not reported)	6.0 [1.5– 12.8] h/day	No difference in intubation, oxygenation, or mortality	No difference in FiO_2 at discharge or serious adverse events
Grieco et al. 2023 (46)	Physiological crossover study	15 ARDS patients on HFNO; P/F <200, SpO_2 <92%, $PaCO_2$ <45 mmHg	66 [62–75]	HFNO 60 L/min; FiO_2 titrated to SpO_2 92–98%	1 h supine → 2 h prone → 1 h supine (single session)	P/F improved from 123 [111–155] to 191 [125–217] mmHg; p<0.001	By EIT: reduced strain (0.38 to 0.21; p=0.002) and +279% EELI (p=0.002)
Liu et al. 2024 (47)	RCT	409 COVID-19–related AHRF; Prolonged APP (n=205) vs SC + short APP (n=204)	68 (± 10) vs 69 (± 10)	Standard oxygen, HFNO or NIV; FiO_2 titrated to SpO_2 >90%	Prolonged: 12 h/day; Control: 5 h/day	Reduced intubation and 28-day mortality: RR 0.62 [0.42–0.90]	More hospital-free days on day 28: 11 (± 9) vs 9 (± 8)

Randomized clinical trials and physiological studies evaluating awake prone positioning (APP) in non-intubated patients with COVID-19–related acute hypoxemic respiratory failure. Studies differ in population severity, respiratory support modality, and daily exposure to prone positioning. Primary outcomes include intubation, mortality, or physiological improvement in oxygenation, while secondary outcomes include resource use, complications, and regional lung mechanics assessed by electrical impedance tomography (EIT). When available, results are reported as mean (± SD) or median [interquartile range]. **Abbreviations:** AHRF, acute hypoxemic respiratory failure; APP, awake prone positioning; ARDS, acute respiratory distress syndrome; C-ARDS, COVID-19–related acute respiratory distress syndrome; COT, conventional oxygen therapy; CRRT, continuous renal replacement therapy; ECMO, extracorporeal membrane oxygenation; EELI, end-expiratory lung impedance; EIT, electrical impedance tomography; FiO_2, fraction of inspired oxygen; HF, heart failure; HFNO, high-flow nasal oxygen; ICU, intensive care unit; IMV, invasive mechanical ventilation; IQR, interquartile range; LOS, length of stay; NIV, noninvasive ventilation; $PaCO_2$, arterial partial pressure of carbon dioxide; PAPP, prolonged awake prone positioning; PEEP, positive end-expiratory pressure; P/F, PaO_2/FiO_2

ratio; PP, prone position/prone positioning; PS, pressure support; RCT, randomized controlled trial; RR, relative risk; SAPP, short awake prone positioning; S/F, SpO_2/FiO_2 ratio; SP, supine position; SpO_2, peripheral oxygen saturation; ST, standard therapy.

Supplementary Table S2. Prone positioning in patients with ARDS, including those supported with ECMO: clinical studies evaluating duration, timing, physiological effects, and outcomes.

Author/ Year	Study Design	Population	Age (Years)	ARDS Severity	Ventilatory Strategy	Prone Positioning Exposure	Primary Outcome	Secondary Outcome
Giani et al. 2021 (55)	Multicenter cohort study	240 ARDS patients on ECMO (107 prone; 133 supine)	48-49 (±13)	Severe ARDS	VT adjusted to maintain ΔP 10–12 cmH_2O; RR ≈20/min; ECMO blood flow 3.7–3.8 (±0.7–0.8) L/min; sweep gas flow 6.5 (±2.5) L/min	15 [12–18] h/session	Prone vs supine: ECMO duration 16 [11–30] vs 10 [6–18] days ($p < 0.0001$); time alive on ECMO 14 [10–24] vs 10 [6–16] days ($p = 0.0011$)	ICU LOS: 35 [21–50] vs 26 [15–51] days ($p = 0.017$); hospital mortality: 34% vs 49.6% ($p = 0.017$)
Page et al. 2022 (57)	RCT	52 C-ARDS patients randomized 1:1 (16 h PP vs 24 h PP)	16 h: 62 (±12); 24 h: 64 (±14)	Severe ARDS	VT 6 ml/kg PBW; PEEP: 11.8 (±2.1) vs 12.8 (±2.7) cmH_2O	16 h vs 24 h	No significant difference in PaO_2/FiO_2 ratio	No significant differences in ICU LOS or duration of IMV
Fossali et al. 2022 (51)	Physiological crossover study	21 C-ARDS patients	67 [61–72]	Severe ARDS	PRVC, VT 6–8 ml/kg PBW, PEEP 10 cmH_2O, FiO_2 94–98%, EIT monitoring	36 [16–72] h	Prone vs supine ($p < 0.001$): reduced ventral dead space fraction (11.3 [3.7–19.0] vs 1.5 [0.4–6.0]); reduced ventral VT distribution (53 ± 8% vs 40 ± 11%); increased dorsal VT distribution (47 ± 9% vs 60 ± 11%)	PaO_2 increased from 85 (±21) to 142 (±90) mmHg ($p < 0.001$)
Camporota et al. 2023 (60)	Observational Study	376 patients (156 ARDS, 220 C-	ARDS: 63 [52–71]; C-	Moderate-Severe ARDS	ARDS: VT 6.1 [5.9–6.6] ml/kg PBW; C-ARDS:	ARDS: 28 [16–48] h; C-	ARDS vs C-ARDS ($p < 0.001$): ΔP reduction −12% vs 0%;	ICU LOS: 18 vs 22 days

		ARDS), all receiving ≥1 PP session	ARDS: 62 [56–70]		6.51 [6.0–7.6] ml/kg PBW	ARDS: 62 [30–93] h	ventilatory ratio reduction −7% vs −2%	
Schmidt et al. 2023 (PRONECMO Trial) (54)	RCT	170 patients on VV-ECMO randomized 1:1 to prone or supine	Prone: 52 [44–60]; Supine: 50 [41–58]	Severe ARDS	Prone: VT 3.0 [2.0–4.3] ml/kg PBW, ΔP 14 [11–15] cmH_2O; Supine: VT 3.1 [2.3–3.9], ΔP 14 [11–14]	≥4 sessions of 16 h	No difference in ECMO weaning at 60 days (p = 0.64)	No significant differences in ECMO duration, IMV duration, respiratory mechanics, ICU or hospital LOS
Okin et al 2023 (56)	Retrospective Cohort Study	267 COVID-19 patients (110: 16 h PP; 157: 24 h PP)	16 h: 60 [51–74]; 24 h: 63 [52–70]	Moderate-Severe ARDS	VT ≈6.0 ml/kg PBW; PEEP 12 [10–14] cmH_2O	1–4 days	24 h PP associated with lower 30-day mortality (aHR 0.475; p < 0.001) and 90-day mortality (aHR 0.638; p = 0.006)	No difference in time from intubation, ICU LOS, hospital LOS, or ventilator-free days
Simpkin et al. 2025 (59)	Prospective cohort study	3,131 patients: never proned (n=1,482); proned after 48 h (n=615); proned within 48 h (n=1,034)	G1: 58 ± 16; G2: 58 ± 14; G3: 57 ± 13	Moderate-Severe ARDS	Not reported	Not reported	Proning within 48 h associated with lower 28-day mortality (HR 0.82, 95% CI 0.68–0.98; p = 0.029) and 90-day mortality (HR 0.81, 95% CI 0.68–0.96; p = 0.017) vs never proned	No significant differences in PaO_2/FiO_2 ratio between groups

This table summarizes clinical studies evaluating the use of prone positioning in patients with moderate to severe ARDS, including cohorts with venovenous extracorporeal membrane oxygenation (VV-ECMO) support. The studies encompass randomized controlled trials, prospective and retrospective observational cohorts, and physiological investigations. Data includes patient characteristics, ARDS severity, ventilatory strategies, duration of prone positioning sessions, and major clinical and physiological outcomes. Primary outcomes reflect study-specific endpoints, whereas secondary outcomes include relevant clinical, respiratory mechanics, and survival-related variables. When available, results are reported as mean (± SD) or median [interquartile range]. **Abbreviations:** ARDS, acute respiratory distress syndrome; aHR, adjusted hazard ratio; CI, confidence interval; C-ARDS, coronavirus disease 2019–related acute respiratory distress syndrome; DP, driving pressure; ECMO, extracorporeal membrane oxygenation; EIT, electrical impedance tomography; FiO_2, fraction of inspired oxygen; HR, hazard ratio; ICU, intensive care unit; IMV, invasive mechanical ventilation; IQR, interquartile range; LOS, length of stay; PaO_2, arterial partial pressure of oxygen; PBW,

predicted body weight; PEEP, positive end-expiratory pressure; P/F, PaO_2/FiO_2 ratio; PP, prone position or prone positioning; PRVC, pressure-regulated volume control; RCT, randomized controlled trial; RR, respiratory rate; SD, standard deviation; VT, tidal volume; VV-ECMO, venovenous extracorporeal membrane oxygenation.

Supplementary Table S3. Summary of randomized and observational studies evaluating driving pressure–guided and ultra-protective ventilation strategies, and extracorporeal support in patients with ARDS.

Authors/ Year	Study Design	Population	Age (years)	ARDS Severity	Ventilatory Strategy/ΔP	Extracorporeal Support	Primary Outcome	Secondary Outcome
Romano et al. 2020 (66)	RCT	31 ARDS (mild–moderate), ΔP-limited vs ARDSNet	45 (±18) vs 52 (±15)	Mild–moderate	ΔP-limited: Pplat 24–25 cmH_2O; baseline ΔP ≈15 cmH_2O	None	Greater ΔP reduction day 1→3 in ΔP group (15.3→10.6 vs 15.1→13.9 cmH_2O; p<0.001)	No difference in 28-day mortality, ICU LOS, or VFD
McNamee et al. 2021 (68)	RCT	412 moderate–severe ARDS; LPV+$ECCO_2R$ vs LPV	59	Moderate–severe	LPV 6 ml/kg vs ~3 ml/kg PBW; ΔP 15 vs 16 cmH_2O	$ECCO_2R$ (350–450 mL/min; sweep 10 L/min)	No difference in 90-day mortality	Fewer VFD with $ECCO_2R$ (7.1 vs 9.2; p=0.02); no sig. difference in oxygenation
Guervilly et al. 2022 (69)	RCT	38 severe ARDS on VV-ECMO; ULV vs UPV	56 vs 57	Severe	ULV vs UPV; ΔP 16 vs 17 cmH_2O	VV-ECMO	No difference in IL-1, IL-6, SP-D	TNFα, IL-8, VEGF decreased only in ULV; MP lower in ULV (2.4 vs 5.4 J/min; p=0.003)
Nijbroek et al. 2022 (67)	Observational Cohort	903 COVID-19 ARDS; UPV vs LPV	65 [57–72]	Moderate–severe	UPV vs LPV	None	Higher 28-day mortality with LPV (31.7% vs 23.1%; p=0.005)	Lower MP and Crs in UPV; greater NMB

								use (57.1% vs 43.8%; p<0.001)
de Haro et al. 2023 (63)	RCT	98 patients at risk for ARDS; LPV vs intermediate VT	66 [55–73]	Mild–moderate	4–6 vs 8–10 ml/kg PBW; ΔP 12 [9–14]	None	No difference in ARDS development	Higher 28- and 90-day mortality in LPV group
Monet et al. 2024 (64)	Prospective cohort	41 ARDS on $ECCO_2R$	60 [48–65]	Moderate–severe	≤3 vs >3 ml/kg PBW; ΔP 14 vs 20 cmH_2O	$ECCO_2R$ (low vs high flow)	90-day survival: 63% vs 18% (p=0.0004)	Lower MP after 24 h in success group; high-flow $ECCO_2R$ associated with success
Rixecker et al. 2025 (65)	Observational cohort	1137 COVID-19 ARDS on ECMO	41–70 (median range)	Severe	UPV + PEEP 10–16 cmH_2O	ECMO	Overall mortality 78%	No ΔP difference between survivors vs non-survivors; survivors had higher Crs at day 14
Maia et al 2025 (STAMINA) (62)	RCT	198 moderate-severe CAP-ARDS	63 [47 - 73]	Moderate–severe	ΔP-limited + PEEP titration vs low-PEEP LPV	None	No difference in VFD at day 28	Mortality 66% vs 53% (p=0.13)

Studies are summarized according to design, patient population, ventilatory strategy, use of extracorporeal support, and principal clinical and physiological outcomes. When available, results are reported as mean (± SD) or median [interquartile range]. **Abbreviations:** ARDS, acute

respiratory distress syndrome; C-ARDS, COVID-19–related ARDS; CAP-ARDS, community-acquired pneumonia–related ARDS; RCT, randomized controlled trial; $ECCO_2R$, extracorporeal carbon dioxide removal; ECMO, extracorporeal membrane oxygenation; VV-ECMO, veno-venous ECMO; UPV, ultra-protective ventilation; ULP/ULV, ultra-lung-protective ventilation; LPV, lung-protective ventilation; ITV, intermediate tidal volume ventilation; VT, tidal volume; PBW, predicted body weight; PEEP, positive end-expiratory pressure; Pplat, plateau pressure; ΔP, driving pressure; MP, mechanical power; Crs, respiratory system compliance; $PaCO_2$, arterial partial pressure of carbon dioxide; PaO_2, arterial partial pressure of oxygen; FiO_2, fraction of inspired oxygen; VFD, ventilator-free days; ICU, intensive care unit; LOS, length of stay; NMB, neuromuscular blockade; TNFα, tumor necrosis factor alpha; VEGF, vascular endothelial growth factor; SP-D, surfactant protein D.

Supplementary Table S4. Physiological and clinical studies evaluating PEEP titration strategies and advanced monitoring in patients with ARDS.

Authors/ Year	Study Design	Population	Age (Years)	Study Intervention	Primary Outcome	Secondary Outcome
Salem et al. 2020 (92)	RCT	60 ARDS patients randomized 1:1 to LUS-guided PEEP (G1) or FiO_2-based PEEP (G2)	G1: [20–80]<br>G2: [18–70]	Lung-protective ventilation (VT 4–8 mL/kg PBW)	G1 vs G2: Higher optimal PEEP (10 vs 8 cmH_2O) and higher PaO_2/FiO_2 ratio (266 ± 44.5 vs 233 ± 53.9; $p < 0.001$)	Higher static compliance (54.8 ± 6.6 vs 45.9 ± 3.8 mL/cmH_2O; $p < 0.001$); more ventilator-free days (23 [0–22] vs 20 [0–22]; $p < 0.001$); lower 28-day mortality (6.7% vs 30%; $p = 0.041$)
Grieco et al. 2023 (80)	Physiological cohort study	20 patients with moderate-to-severe COVID-19 ARDS	63 [56-74]	Decremental PEEP trial (15, 13, 10, 8, 5 cmH_2O)	PEEP 15 to 5 cmH_2O: Decrease in EELV (2,464 [1,958–3,049] to 1,570 [1,101–1,921] mL; $p < 0.001$); decrease in strain (0.36 [0.31–0.41] to 0.15 [0.12–0.17])	Decrease in FRC + recruited volume (1,719 [1,439–2,230] to 1,357 [962–1,698] mL); strong correlation between recruitment-to-inflation ratio and respiratory system compliance
Jimenez et al. 2023 (81)	RCT	12 ARDS patients	61 [48-68]	EIT-guided PEEP titration vs PEEP/FiO_2 tables	EIT strategy reduced mechanical power (−2.50 ± 3.70 vs +1.87 ± 1.61 J/min; $p = 0.002$) and elastic–dynamic power (−1.13 ± 1.66 vs +0.48 ± 0.88 J/min; $p = 0.027$)	Not reported

Borlino et al. 2024 (84)	Cohort study	23 ARDS patients (10 low recruiters, 13 high recruiters)	70 [61–75]	Assessment of recruitment-to-inflation ratio at PEEP 4 and 14 cmH_2O	Increasing PEEP reduced cardiac index (3.3 [2.5–4.1] to 2.9 [2.5–3.7] L/min/m²; $p = 0.02$)	In low recruiters: increased RV/LV end-diastolic area ratio and reduced TAPSE; greater reduction in TAPSE/SPAP ratio compared with high recruiters
Mauri et al. 2024 (89)	RCT	30 patients with moderate-to-severe ARDS	63.6 (± 14.2)	PEEP titration guided by EIT–ΔPL vs PEEP/FiO_2 table during PSV	EIT–ΔPL strategy selected higher PEEP (10 [8–12] vs 8 [5–10] cmH_2O; $p = 0.021$) and reduced dynamic ΔPL (15.1 ± 5.1 vs 16.8 ± 6.3 cmH_2O; $p < 0.001$	Reduced inspiratory effort (PTPmus 123.6 ± 46.2 vs 158.8 ± 65.6; $p < 0.001$) and lower P0.1
Lambour et al. 2025 (79)	Physiological cohort study	34 ARDS patients	61 (± 13)	Recruitment maneuver to PEEP 40 cmH_2O followed by decrement to PEEP 25 cmH_2O	Post- vs pre-recruitment: Increased CO, SV, and CI; reduced LVEDV (all $p < 0.001$)	Improved respiratory system compliance and ventilatory ratio (all $p < 0.001$)
Bello et al. 2024 (83)	RCT	16 patients with $PaO_2/FiO_2 < 200$ mmHg and $\Delta Pes > 10$ cmH_2O	75 [69-78]	PSV and PCV at PEEP 5 and 15 cmH_2O with EIT monitoring	No difference in inspiratory effort or driving pressure between PEEP levels	Higher PEEP reduced regional tidal impedance variation and increased aerated lung volume in both PSV and PCV
Sutherasan et al. 2025 (77)	Prospective physiological study	21 patients with moderate-to-severe ARDS	61 (± 20)	Recruitment maneuver followed by decremental PEEP titration with EIT	Transpulmonary pressure decreased with decremental PEEP ($p < 0.001$)	Progressive increase in EIT-assessed lung collapse (0% at PEEP 16 to 21% at PEEP 8 cmH_2O; $p < 0.001$); no relevant hemodynamic changes

Tsolaki et al. 2025 (93)	Prospective study	106 patients with COVID-19 ARDS	65.2 (± 1.2)	Lung-protective ventilation with PEEP reduction from 13.0 ± 0.3 to 9.5 ± 0.3 cmH_2O	PEEP reduction improved right ventricular function: increased RVEF and reduced RVEDV and RVESV	Not reported

This table summarizes randomized and observational studies assessing different strategies for positive end-expiratory pressure (PEEP) titration in patients with acute respiratory distress syndrome (ARDS), including approaches guided by lung ultrasound (LUS), electrical impedance tomography (EIT), transpulmonary pressure (ΔPL), recruitment-to-inflation ratio, and standard PEEP/FiO_2 tables. Studies report the physiological effects of PEEP adjustments on lung mechanics, regional ventilation, lung volumes, respiratory effort, and cardiopulmonary interactions, as well as selected clinical outcomes. Data are presented as mean (± standard deviation) or median [interquartile range], as reported in the original studies. **Abbreviations:** ARDS, acute respiratory distress syndrome; AOP, airway opening pressure; CI, cardiac index; CO, cardiac output; Crs, respiratory system compliance; C-ARDS, coronavirus disease 2019–related ARDS; ΔP_L, transpulmonary driving pressure; ΔP, driving pressure; EELV, end-expiratory lung volume; EIT, electrical impedance tomography; FiO_2, fraction of inspired oxygen; FRC, functional residual capacity; IMV, invasive mechanical ventilation; LRM, lung recruitment maneuver; LUS, lung ultrasound; LVEDV, left ventricular end-diastolic volume; MP, mechanical power; PaO_2/FiO_2, ratio of arterial oxygen partial pressure to fraction of inspired oxygen; PBW, predicted body weight; PCV, pressure-controlled ventilation; PEEP, positive end-expiratory pressure; PSV, pressure support ventilation; PTPmus, pressure–time product of respiratory muscles; R/I ratio, recruitment-to-inflation ratio; RVEDV, right ventricular end-diastolic volume; RVEF, right ventricular ejection fraction; RVESV, right ventricular end-systolic volume; SPAP, systolic pulmonary artery pressure; SV, stroke volume; TAPSE, tricuspid annular plane systolic excursion; VT, tidal volume.

Supplementary Table S5. Neuromuscular blockade in ARDS: physiological and clinical studies.

Authors/ Year	Study Design	Population	Age (Years)	ARDS Severity	NMB Duration	Ventilatory Strategy/ΔP	Main Outcomes
Guervilly et al. 2017 (94)	RCT	24 patients randomized 1:1 to NMB or control	NMB: 72 [63–79]; Control: 60 [52–75]	Moderate ARDS	Cisatracurium: 15 mg IV bolus followed by continuous infusion 37.5 mg/h	Lung-protective ventilation	Compared with control, NMB significantly increased inspiratory and expiratory transpulmonary pressure (8.7 ± 3.3 vs 5.7 ± 3.6 cmH_2O; and 1.4 ± 2.7 vs −1.8 ± 3.5 cmH_2O, respectively; p = 0.01) and reduced plateau pressure (20 [17–21] vs 22 [19–26] cmH_2O; p = 0.05).
Courcelle et al. 2020 (99)	Observational Study	407 patients with COVID-19 ARDS: ≤2 days NMB (G1) vs >2 days NMB (G2)	G1: 63 [56–71]; G2: 65 [55–72]	Moderate-Severe ARDS	≤2 days vs >2 days	Lung-protective ventilation; ΔP: 11 [9–13] vs 12 [10–14] cmH_2O	No difference in extubation at day 28. Compared with G2, G1 had lower prone positioning use (65% vs 90%, p < 0.001), lower ICU mortality (25% vs 39%, p = 0.001), and fewer days of mechanical ventilation among ICU survivors (15 [9–26] vs 20 [13–32] days; p = 0.003).
Li Bassi et al. 2022 (98)	Comparative observational study with matched cohorts	1953 COVID-19 ARDS patients (1711 LPV only; 243 LPV + NMB); 210 matched per group	LPV: 61.2 ± 14.2; LPV + NMB: 59.4 ± 12.1	Moderate-severe ARDS	Short course: 48 h up to 3 days, initiated within 48 h of IMV	LPV; PEEP 11–12 cmH_2O; median ΔP 22 cmH_2O	Ninety-day mortality increased with longer NMB exposure (30% after 2 days; 50% after 3 days). Ventilator-free days at day 28: 16 (LPV) vs 25 (LPV + NMB), p = 0.055.

Lee at al. 2022 (103)	Observational Study	129 COVID-19 ARDS patients: 98 received NMB, 31 did not	69 [62–78]	25: Mild<br>55: Moderate<br>49: Severe	Median duration: mild 5 [4–9] days; moderate 5 [4–8] days; severe 8 [4–15] days	VT 6.8–7.3 mL/kg IBW; ΔP: mild 14 [11–16], moderate 12 [11–15], severe 14 [13–18] cmH_2O	Compared with no NMB, NMB was associated with higher ICU mortality (58% vs 31%, p = 0.006), higher hospital mortality, higher weaning rate (66% vs 42%, p = 0.02), more ventilator-free days at day 28 (9.0 ± 9.8 vs 5.7 ± 8.9), and lower tracheostomy rate (28% vs 50%, p = 0.02).
Rollinson et al. 2024 (97)	Observational study	220 COVID-19 ARDS patients: 56 prone positioning only, 164 NMB + prone positioning	54 (±13)	Moderate-severe ARDS	Cisatracurium for 48 h (dose not reported)	VT 7 mL/kg PBW; median PEEP 11–12 cmH_2O; median ΔP 14–15 cmH_2O	Mean PaO_2/FiO_2 during first prone session was higher in the NMB group (208 ± 63 vs 161 ± 66 mmHg). Oxygenation remained consistently higher in the NMB group across four prone sessions.

This table summarizes randomized and observational studies evaluating the physiological and clinical effects of neuromuscular blockade (NMB) in patients with acute respiratory distress syndrome (ARDS), including COVID-19–related ARDS. Studies are ordered chronologically. Data are presented as mean ± standard deviation, median [interquartile range], or number (%), as reported in the original publications. Ventilatory variables refer to values measured during controlled mechanical ventilation. Outcomes include physiological variables, oxygenation, ventilator settings, and clinical endpoints. When applicable, comparisons are reported between patients receiving NMB and control groups, or according to NMB exposure duration. **Abbreviations:** ARDS, acute respiratory distress syndrome; CI, confidence interval; COVID-19, coronavirus disease 2019; ΔP, driving pressure; ICU, intensive care unit; IMV, invasive mechanical ventilation; IBW, ideal body weight; LPV, lung-protective ventilation; MV, mechanical ventilation; NMB, neuromuscular blockade; PaO_2/FiO_2, ratio of arterial oxygen partial pressure to inspired oxygen fraction; PBW, predicted body weight; PEEP, positive end-expiratory pressure; PL, transpulmonary pressure; PP, prone positioning; RCT, randomized controlled trial; VFD, ventilator-free days; VT, tidal volume.
